# Disentangling What We Know About Microbes and Mental Health

**DOI:** 10.3389/fendo.2019.00081

**Published:** 2019-02-15

**Authors:** Gregor Reid

**Affiliations:** ^1^Canadian R&D Centre for Human Microbiome and Probiotics, Lawson Health Research Institute, London, ON, Canada; ^2^Department of Microbiology and Immunology, and Surgery, Western University, London, ON, Canada

**Keywords:** gut, *Lactobacillus*, probiotic, Hadza hunter-gatherers, brain

## Abstract

Much has been written in recent years about the gut-brain axis. Exciting pilot studies suggest probiotic applications to the gut can reduce anxiety and depression via the vagus nerve. But not to diminish such findings, much still needs to be considered, including the fact that the vagus nerve links to many other body sites that also host a microbiome. Questions remain that touch the core of being human: (i) Do our microbes influence happiness and to what extent? (ii) What components of the gut microbiota and their function, including as it relates to mental health, are critical and how do they differ between agile, fit hunter gatherers and obese westerners or Danes described as the happiest people on the planet? (iii) What role do environmental pollutants play in this microbes-host ecosystem? While approaching life from a reductionist perspective has a long history in science, we need to try to interrogate these health and disease issues from a wider perspective. For verification of a link between the gut microbiota and brain, and to test new therapies, human studies are needed, and are long overdue.

## Introduction

There is arguably no better place to witness first-hand the impact that diet and microbes have on health and the way the brain functions, than in developing countries of Africa. The parasites and pathogens that can debilitate Westerners have long been known, and the resilience of the local Africans to many of these agents has played its part in the evolution of the hygiene hypothesis, understanding chronic diseases, and creation of a range of vaccines, including against the Ebola virus (1). Having been party to the study of Hadza hunter gatherers (2) and noting that they too can live to be elderly, it is clearly not a simple task to unmask the role of diet, microbes, and lifestyle in longevity.

It is challenging to dissect the effects of microbes on brain function. To date, the focus has been on pain, anxiety, depression (3), centered in the amygdala within the limbic system, and linked through the vagus nerve. These studies have not examined the more positive emotions of happiness, drive, and contentment that often appear to be in high abundance among Africans, despite their often-dire predicament.

This paper will explore the potential interplay between food, microbes and lifestyle, and challenge researchers to verify rodent research in humans.

## Trying to Understand the Microbiota Through Retracing Human Evolution

While the environments of Canada and east Africa certainly differ to a large extent, as do diets for the majority of citizens, there are also commonalities. For example, the vaginal microbiota shares the same dominant *Lactobacillus* strains across continents, likely indicating their contribution to successful reproduction (4).

In search of increasing our understanding of microbiome evolution, studies have been performed on ancient hunter gatherers in Africa and South America. While they are not free from influence by modern society, some of the habits remain intact. A recent study showed that the Hadza gut microbiota is influenced by seasonal dietary intake, varying from meat to certain fruits (2). The resultant increase in diversity is likely a hallmark of how humans used to consume food, except that Westernized societies have access to clean water, unlike the Hadza who live in the Central Rift Valley in Tanzania, have significant hand-fecal (human and animal) exposure, and drink untreated water. Indeed, the introduction of un-milled maize to individuals normally consuming baobab, roots, berries, and honey significantly changed their gut microbiota (5).

But, how such seasonality and microbial patterns influence mood has not been studied. The ability to cover great distances in bare feet, hunting animals, securing a temporary home, and providing for women and offspring cannot only be a measure of testosterone and Y chromosomes. If that were the case, the same drive would arguably be present in Hadza hunter gatherers who have assimilated into the lifestyles of Westernized Africans, consuming processed foods, using pharmaceutical agents, and receiving vaccines. Indeed, testosterone levels in one group of Hadza were found not to change with age, and not to be linked to total energy expenditure (6). The loss of *Treponema* sp. and acquisition of *Bifidobacterium* sp. has been reported in Westernized hunter gatherers, the *Treponema* being more suitable for degradation of unrefined plants, and the bifidobacteria better suited to utilizing gluco- and galacto-based saccharides of Western foods (7). So, diet and lifestyle do alter the microbiota, but this does not prove a correlation with changes in energy and happiness.

Norway, Denmark, and Sweden are ranked in the category of happiest places to live (8), based upon governance, personal freedom, opportunity, education, safety, and healthcare. Suicide rates are around 11 per 100,000, yet approximately half Norwegians will suffer depression at some point in their life (9). Are rates of depression a more suitable measure of happiness and contentment, and if so, what about depression amongst hunter gatherers? Based on observations from Papua New Guinea reporting no depression in hunter gatherers, an explanation was consumption of omega-3 fatty acids, access to regular sunlight, exercise, social interaction, and healthier sleep (10). So, could a supplement of certain probiotic strains, vitamin D, and omega-3 fatty acids reduce depression rates in countries where people have good access to employment, housing, education, and healthcare? These are questions that require investigation.

## The Gap Between Mouse Studies and Humans

Much has been made of the link between autism and the gut microbiota (11). One study using a mouse model suggested that *Bacteroides fragilis* therapy had potential to resolve behavioral symptoms in autistic humans (12). Whether an already unnatural mouse develops autism does not then translate into a therapeutic breakthrough for autistic children, particularly when analytical flaws in the study were exposed by others (13). Such studies lead to internet blogs that distort the reality and raise hope for a cure in parents. Although scientists have little control over how lay people or the media represent their work, the way that conclusions are presented and the rigor of the review process to minimize sensationalizing results have a major impact.

The basis of too many microbiome studies, to date, has been rodent experiments, or observational studies in humans, neither of which prove cause and effect. In the case at hand, parents desperate to help their autistic child might look to fecal microbiota transplant or a *B. fragilis* probiotic that does not exist and will take many years to develop, believing these treatments will alleviate certain behaviors. It is not for me to say that such interventions will or will not work, but there should be a better scientific basis for suggesting they might be effective. Instead, the senior author of the mouse paper (12) published a review in the same journal Cell (14) reiterating the link between the gut microbiota and the brain including autism spectrum disorder as if it was proven in humans, when almost all the citations refer to mouse experiments, and human observations. Indeed, the review itself cites numerous other reviews making the same points based on rodent experiments.

The possibility that vast numbers of microbes in the intestinal tract with the potential to produce compounds that include neurochemicals (15, 16) can influence the brain is compelling. That the vagus nerve is a main conduit for such signaling (17) is suggested by the vastness of innervation and sensory fibers, and by vagotomy studies (18), but other sites especially in the urogenital and respiratory tracts served by this nerve also have microbiotas (19). Indeed, afferent nerves within the vagus innervate almost all visceral organs, and in the airways and lungs, vagal sensory neurons are the major afferent supply and close to 20% terminate there (20). The airway microbiota contains a number of species quite capable of influencing the nervous system (21, 22). These discoveries have led to the concept of a gut-lung axis, influenced by diet and the microbiota (23, 24). The importance of the vagus nerve in the microbe-gut-brain axis (25) has led to exploration of chronically stimulating the nerve to treat Crohn's disease (26). However, vagus nerve stimulation therapy is not without difficulty, and requires a wire with three helical contacts and a one-pin battery, which can potentially lead to infections, delayed arrhythmias, hoarseness, dyspnea, coughing, and vocal cord damage emphasizing the linkage between the nerve and sites other than the gut (27). In studies of the gut-brain access via the vagus nerve, it may prove challenging to deduce the extent to which organisms in the gut vs. other sites linked to the nerve, are impacting the brain. Of note, the contribution of the vagus nerve to monitoring the microbiome is based on marginal evidence apart from a few vagotomy studies, and such animals suffer from impaired gastrointestinal functions. In addition to the vagus nerve, the intestinal mucosa has sensory functions and neurons, endocrine cells, and immune cells which act as detectors (28). This makes it possible for the brain to control important functions such as gut motility.

What has so far been lacking is identification of molecules produced by microbes that are responsible for brain effects. Arguably, the tools are available to identify these in humans through use of various metabolomic and proteomic methods, and access through surgical procedures. Descriptions of what could, and indeed should, be done in humans, has been lacking from the multitude of reviews on the gut-brain axis, with one exception. Hooks et al. (29) recently raised similar salient points as those made here, including the relevance of rodent studies to humans (30), and calling for research that deciphers the complexity of the multipathway systems. Notably, they state “*It could be well-worth working with relevant public health and media experts on how to communicate this exciting body of work responsibly*” [(29), page 27]. They also add to the discussion the potentially important role of environmental factors in brain related illness, an issue that will be discussed below. The impact of microbial metabolites on the brain is unclear. Only a few G protein-coupled transmembrane molecular sensors have been identified metabolites in the Na_v_1.8-expressing vagal afferents that detect neurotransmitters, hormones, nutrients (31).

Certainly, omics tools could be applied now to patients with multiple sclerosis and other brain related illnesses who are receiving fecal microbiota transplants (FMT) (32) or are taking a variety of probiotics, thereby supporting or rebuking the theories based on rodents. Such research could also be applied to verify other mouse-based studies, for example that suggest FMT from a lean donor can make an obese recipient lose weight (33). Eleven years after this widely cited study, no such conclusive human verification has occurred.

The translation of *in vitro* and animal studies to human experimentation has at least started to occur, with encouraging results against depression by daily ingestion of probiotic bifidobacteria for 6 weeks (34). The selection of the *Bifidobacterium longum* NCC3001 subspecies *longum* strain was from mouse studies 6 years prior showing that ingestion normalized anxiety-like behavior and hippocampal brain derived neurotrophic factor (BDNF) levels (35). The study utilized a range of evaluative tools. Functional MRI analysis showed that reduced responses to negative emotional stimuli in multiple brain areas, including amygdala and fronto-limbic regions, occurred with probiotic treatment compared to placebo. The lack of change in serum inflammatory markers suggested the benefit did not accrue through down-regulation of inflammation. The unchanged levels of neurotrophins and neurotransmitters 5-HT, substance P, and CGRP, would suggest that gut bacteria were not increasing the circulatory levels of at least these three neurotransmitters, even though reductions were noted in urinary levels of phenylacetyglutamine, creatine, 4-cresol sulfate, and trimethylamine-N-oxide. It was no surprise that the fecal microbiota was unchanged, as 16 s rRNA Illumina sequencing is not sensitive enough to detect differences and probiotic strains do not necessarily alter microbial abundances. As to how the probiotic strain was mediating the clinical outcome the answer remains to be found but given the success of this study of 44 subjects, additional trials are warranted, perhaps accompanied by metagenomic, or transcriptomic analyses to see if the bifidobacteria were influencing other microbes or host circuitry.

A challenge for researchers wanting to use microbes to prevent autism spectrum disorder is that interventions will likely need to be tested while the fetus is developing and during early life. This will ethically be more difficult as microbiota manipulation through FMT, probiotics or prebiotics tends not to be target specific, so how the intervention affects other developmental processes will be of concern. On the other hand, if these gut-brain and microbiome-host linkages are true, then the development of every human is already being influenced by a series of microorganisms and in-depth studies during pregnancy and the first year after birth could help to reveal some of the processes. For example, a probiotic strain that improves gut barrier integrity and therefore helps increase adsorption of arachidonic acid, docosahexaenoic acid, and omega-3 polyunsaturated fatty acids, critical for brain growth and cognitive development, could well be tested (36, 37), with implications for later-in-life (38).

As it stands, there are similarities between the challenges facing the microbiome area, still in its infancy, and methodological issues in the field of nutritional epidemiology (39). The author, Ioannidis, viewed most nutritional variables could be correlated with one another if large enough data sets were analyzed. Confounding factors add to the complexity and meta-analyses become weighted averages interpreted by the examiners. As with foods that can contain thousands of chemicals with the most abundant assumed to be responsible for harm or health, so too assumptions are made that abundant microbial taxa are the key influencer. Probing low abundance species and ruling out contamination remain challenges in microbiome studies. As noted above, the propensity of journals to increase manuscript Altmetric scores does not mean the paper or its conclusions represent meaningful results. Ioannidis' call for exploration of new avenues of research and pivotal human trials should drive granting agencies to redirect funds away from often pointless or non-correlative animal experiments.

## Probiotics

As eluded to by Hooks et al. (29), the field of probiotics which faced cynicism in the earlier days of its reemergence (40), is yet again being slated for its lack of usefulness. Unfortunately, the number of people who not understand what probiotics are, and what they are not, is reflected in these commentaries. This is illustrated by a commentary on the Hsiao et al. paper (12) that referred to the *B. fragilis* strain as a probiotic (41), which until proven to confer a health benefit on the host, it is not a probiotic (42). One recent poorly designed and analyzed paper stated in its title a link between small intestinal bacterial overgrowth, probiotics, and metabolic acidosis (43), when no such correlation was found (44). An Israeli group published studies with a product they referred to as “probiotic” but that had failed to meet the well-documented criteria as a probiotic [it did not show a health benefit: (45)]. They tested it in only a handful of healthy subjects and claimed the strains did not colonize and therefore could not be effective (46, 47), even though colonization is not a prerequisite for probiotic strains. In addition, they claimed that probiotics in general might cause harm if used to prevent antibiotic-associated diarrhea, even when no harm was shown. A much more thorough study of almost 400 subjects in a double-blind design, showed that a multi-strain probiotic could in fact correct undesired changes in microbiota composition and function, caused by antibiotic treatments or by cesarean birth (48). This adds to the meta-analysis data showing the benefits of probiotics to prevent antibiotic-associated diarrhea (49).

As Hooks et al. noted, too many microbiome studies, of which the two recent ones are examples (46, 47), “oversell” their limited findings, and in doing so damage a reputable scientific field of probiotics. Such overselling is not exclusive to microbiome research, and of course it can be found across the scientific literature, including for some probiotic studies. But, the point of importance is that such negativity should not lead to cessation of good investigative studies on the potential for microbial interventions to provide a benefit to human mental health.

In addition to the example highlighted above (34), several other excellent studies have shown that probiotic therapy can improve mental health. In a New Zealand study of 423 women, *Lactobacillus rhamnosus* HN001 taken during pregnancy and post-partum significantly reduced depression and anxiety scores compared to placebo (50). In a study investigating neurocognitive impairment in 10 HIV-1 infected patients, 6 months intake of a product containing *Lactobacillus plantarum* DSM 24730, *Streptococcus thermophilus* DSM 24731, *Bifidobacterium breve* DSM 24732, *Lactobacillus paracasei* DSM 24733, *Lactobacillus delbrueckii* subsp. *bulgaricus* DSM 24734, *Lactobacillus acidophilus* DSM 24735, *Bifidobacterium longum* DSM 24736, and *Bifidobacterium infantis* DSM 24737 showed significant improvements in Rey auditory verbal learning test (immediate and delayed recall), Rey-Osterrieth complex figure test (copy immediate and delayed recall), phonological verbal fluency test, Toronto alexithymia scale-20, State-trait anxiety inventory Y-2, and time and weight estimation test scores (51). Interestingly, while one strain of *B. longum* was shown in a small study to improve memory and reduce stress (52), another paired with *L. helveticus* showed no effect on psychological outcome measure (Cohen's d range = 0.07–0.16) (53). Although study designs and subject characteristics differed, it is worth investigating strain to strain variations.

## Environmental and Socio-Economic Confounders

As has been well-documented, mental health, and well-being are affected by a number of factors, not the least of which are socio-economic status and environmental pollutants. Such confounders must be considered in human studies if we are to truly differentiate the role of microbes in alleviating depression, anxiety, and improving treatment of conditions emanating from the brain. Whether this is lack of exposure to coastal waters (54), unemployment (55), being in a household where illness, and death are prevalent (56) or being exposed to small pollutant particles penetrating the blood brain barrier (57), these can influence depression. One study has even suggested that exposure to air pollutants in early life can alter the gut microbiome and increase the risk of various diseases (58). A number of pesticides have been clearly shown to induce neurological disease (59). In some developing countries, sexually transmitted infections and excessive blood or hair mercury levels can be a result of poverty and women trading sex for fish from polluted lakes (60).

In every clinical study, efforts are made to match confounding issues in the active and placebo arms, but perhaps not to the extent of measuring levels of heavy metals and pesticides. These are not only issues for developing countries, but certainly the contamination risk appears to be higher. In a study performed in Mwanza, Tanzania, probiotic *L. rhamnosus* GR-1 yogurt intake correlated with reduced adsorption of mercury, and arsenic in pregnant women consuming contaminated fish from Lake Victoria (61). The lactobacilli were shown to bind to the metals. The subsequent creation of a network of 282 production units across east Africa that make probiotic yogurt made with the GR-1 or *L. rhamnosus* GG generic strain Yoba known also strengthen gut barrier function (62), has the potential to not only reduce toxin uptake and improve nutrient adsorption, but also indirectly improve mental health and well-being. By increasing family income for the producers and along the value chain, the resultant lifestyle, and socio-economic changes could impact rates of depression and anxiety.

This whole-view of society is vital if diseases are to be effectively treated. Unless women are empowered, children are fed, income allows for education and healthcare, and crop losses are minimized, then disease more easily proliferates. A single vaccine or pharmaceutical agent can undoubtedly save lives, but the cost to society in waiting for them to be developed, tested, implemented, and paid for is invariably long; and by the time the shareholders reap the benefits from the consumers directly or countries donating funds for product use, many lives could have been saved or improved in developing countries by simpler remedies borne out of local empowerment. This is not to imply that a probiotic can cure Ebola or malaria, but a practical, affordable probiotic food could indeed be as effective in improving health and well-being, as a capsule imported from the North at a price many-fold higher.

## Concluding Remarks

Health is not a single event. Rather, it is a continuum along which peaks and troughs occur, some more dramatic than others. Microbes are present throughout, and it will be generations before we know how best to program their functionality to impart health and wellness for as long as possible. But, we need scientific rigor in conjunction with translational speed and regulatory flexibility. It need not be driven by large multinationals.

The Hadza have managed without central heating, air conditioning, sugary soda, and automobiles. Some of them live to be very old. Many of them seem happier than the Western scientists who visit them. While fellow humans change the environment in which they live, it may be wise for us to first understand not only the microbes that live in and on the Hadza and the species that surround them, but also how the complexities of human cells, microbes, chemicals, food, and the ecosystem intermingle.

Research into the microbiome has to extend beyond rodent models, as recently shown by a study of over one thousand subjects that identified microbial metabolites correlating with mental quality of life and depression (63). Cancer has been cured many times over in rodents without necessarily doing the same in humans, and clear concerns about animal models and microbiota studies translating to humans have been expressed (64–67). In addition to studies not taking account of the influence of coprophagy (68), many experimental tools and models used to manipulate the microbiome of laboratory rodents are inherently flawed, including the use of antibiotics which have neuro-active and neuro-toxic effects and therefore can influence gut-brain outcomes (69). Concepts can be tested in humans through FMT, probiotics, prebiotics, and dietary alterations. Access to tissue and real-time responses is more feasible now with non-invasive surgery. Such studies will help to clarify conflicting data (70) and make it possible to develop novel approaches to treatment of humans suffering from a range of brain-associated illnesses. Surely, the 17 years it apparently takes to translate science into something that makes a difference to human life (71) is not set in stone?

## Author Contributions

The author confirms being the sole contributor of this work and has approved it for publication.

### Conflict of Interest Statement

GR has consulted on the topic of probiotics for Seed, Acerus Pharma, KGK Synergize, Bayer Consumer Health, Chr Hansen, Metagenics, and Standard Process in the past three years. He receives no licensing fees from the sale of probiotics and has no stock ownership or patents on the topic.
